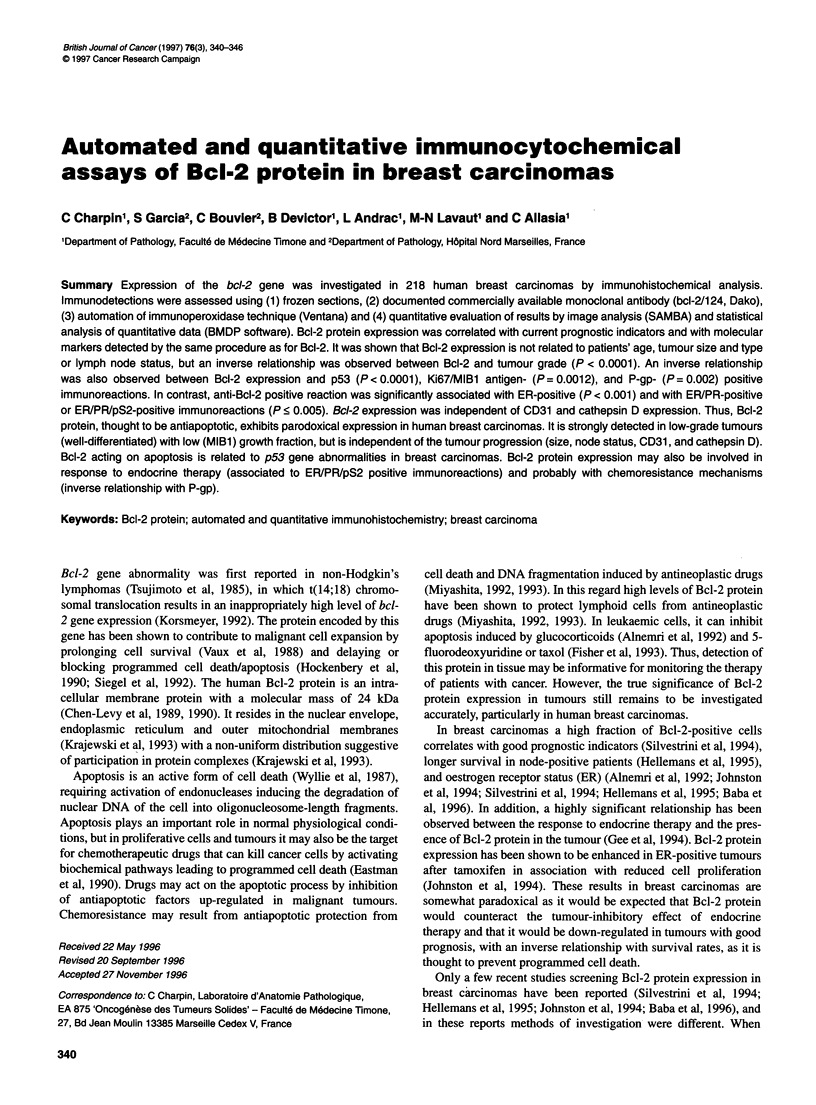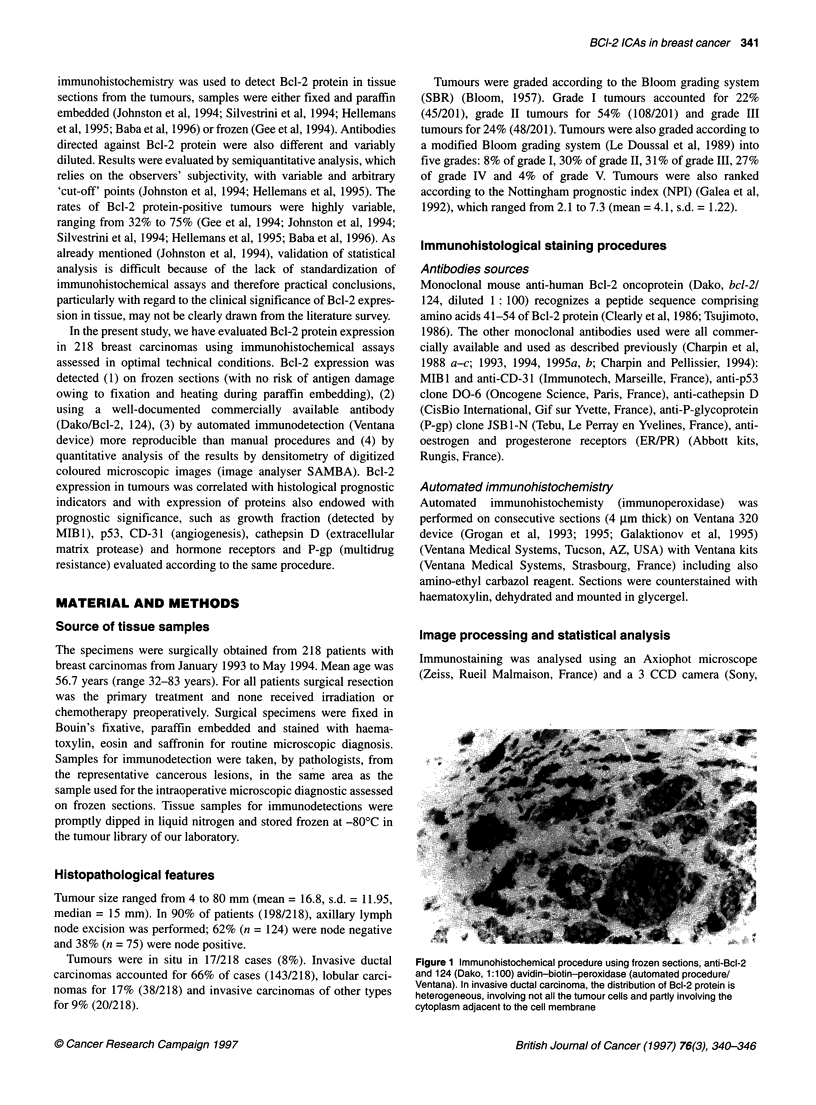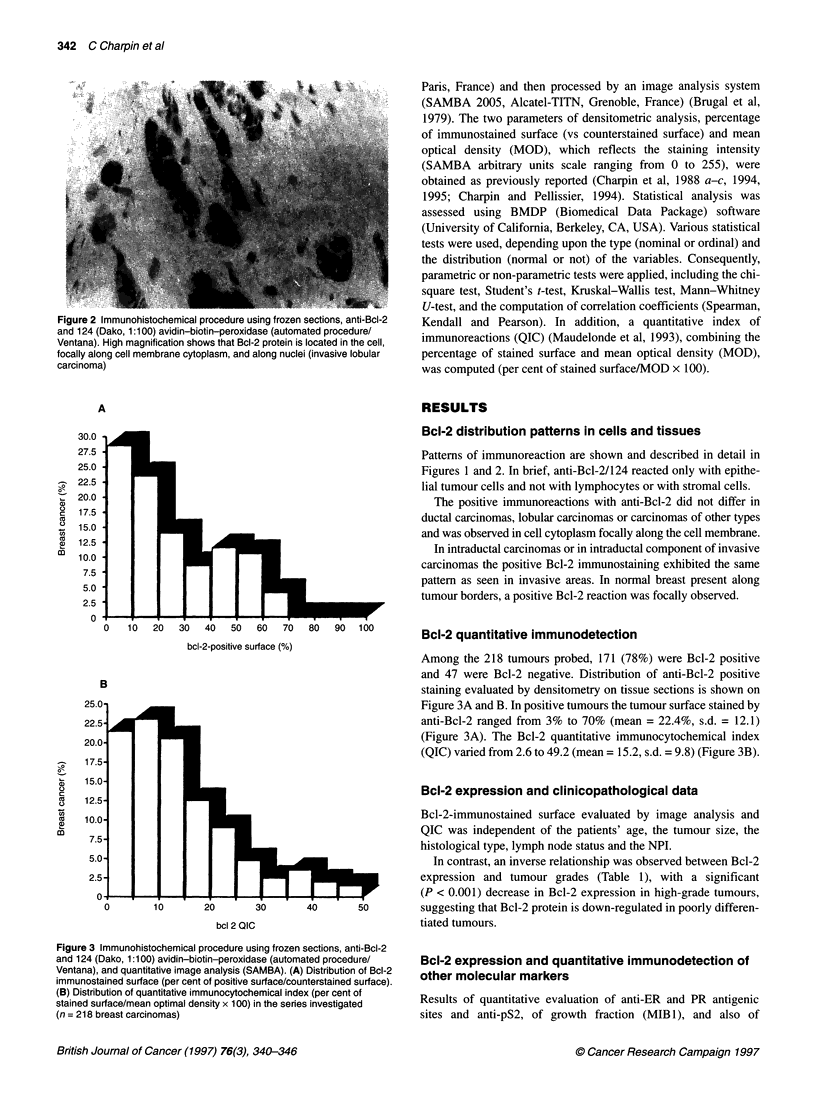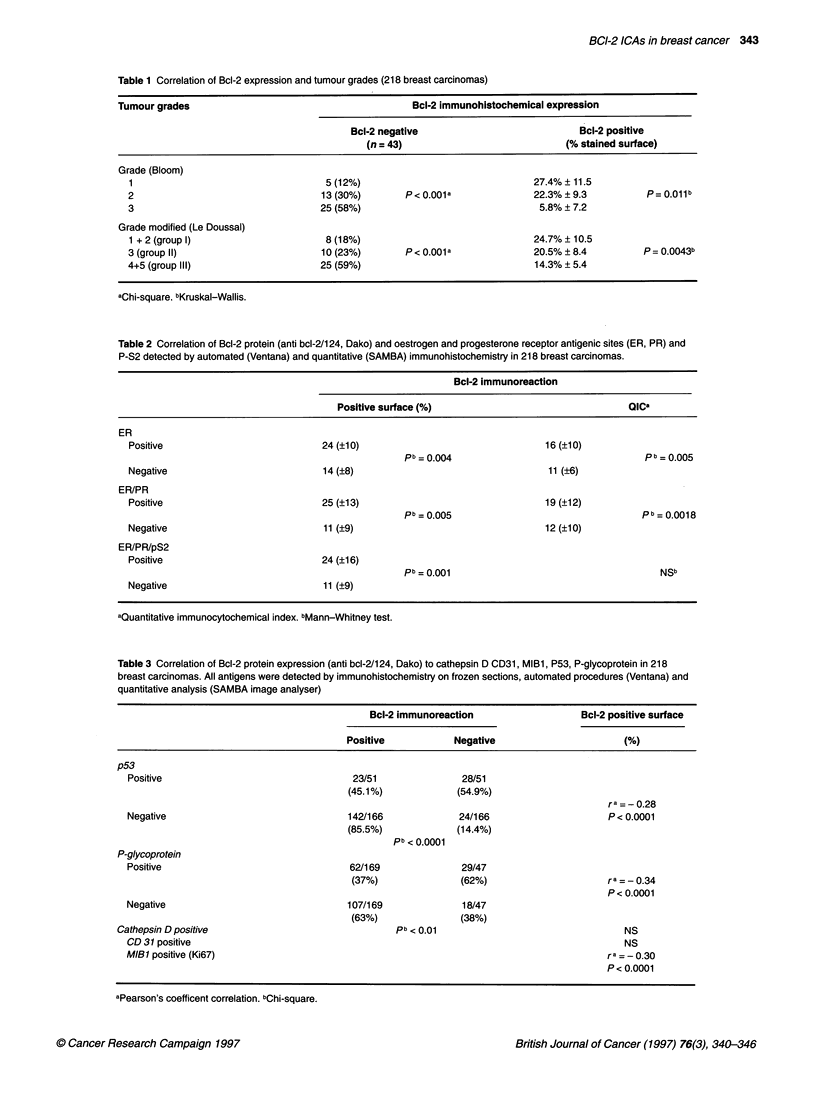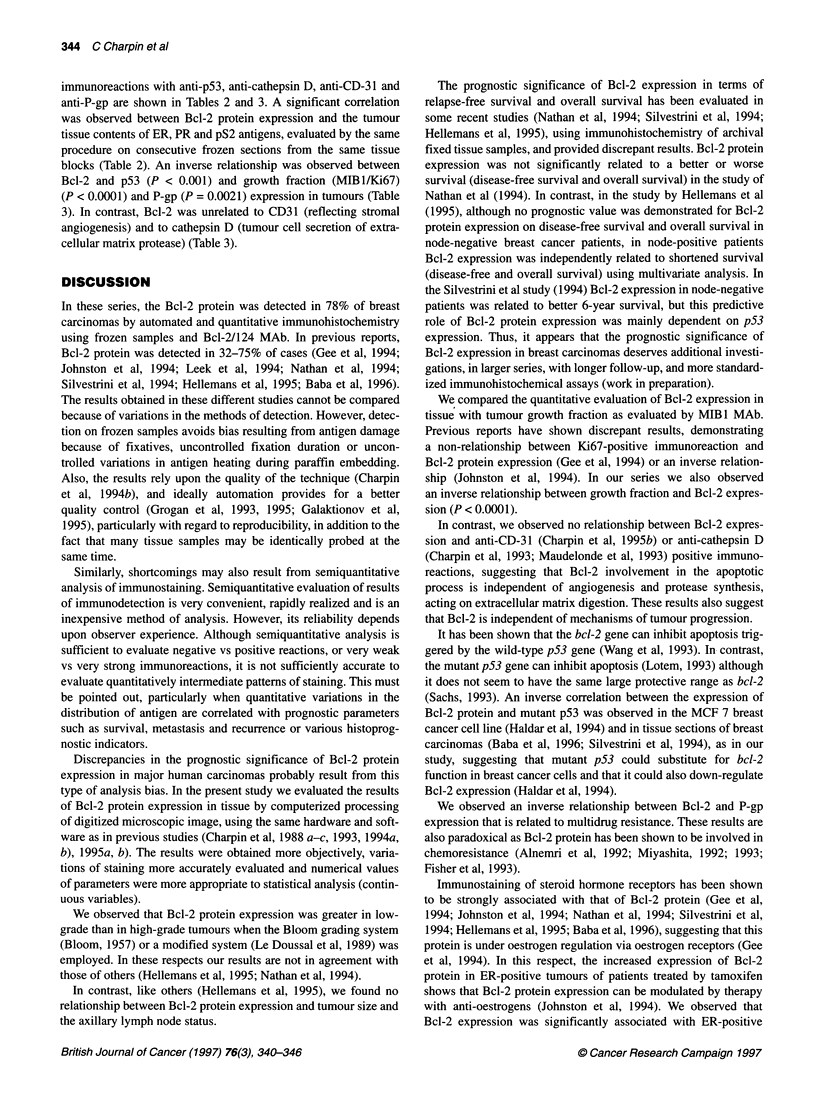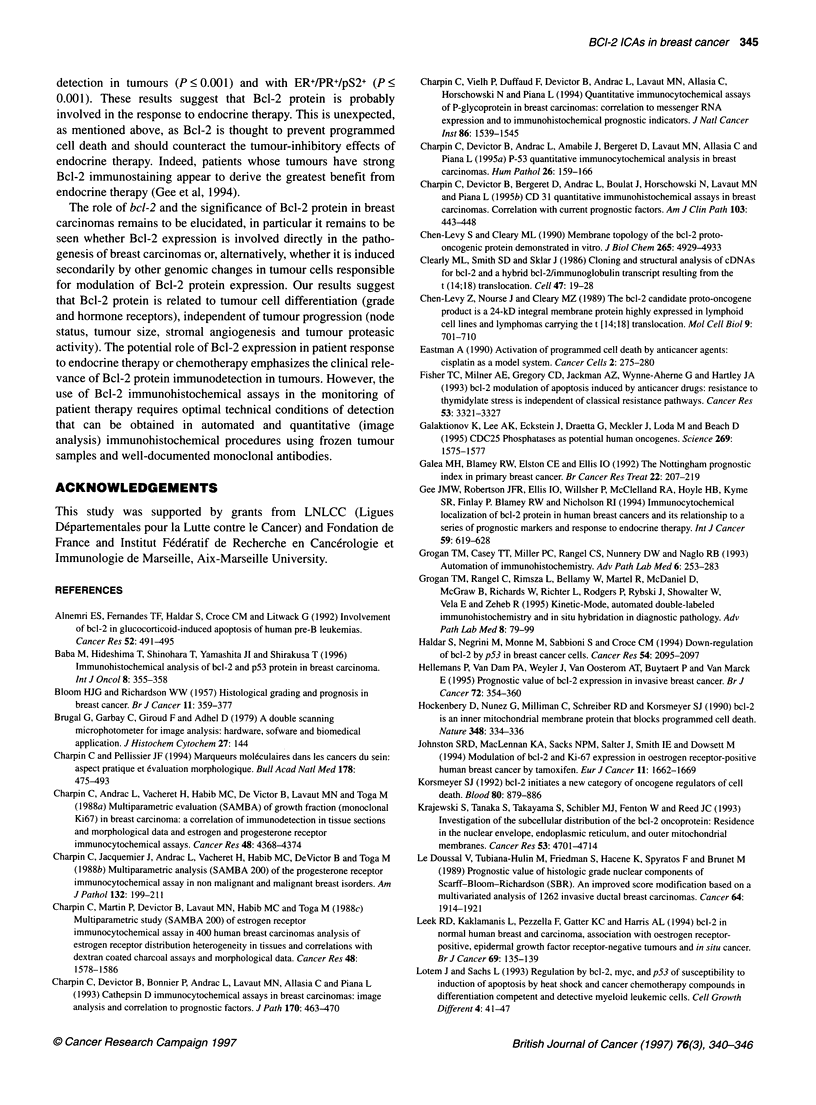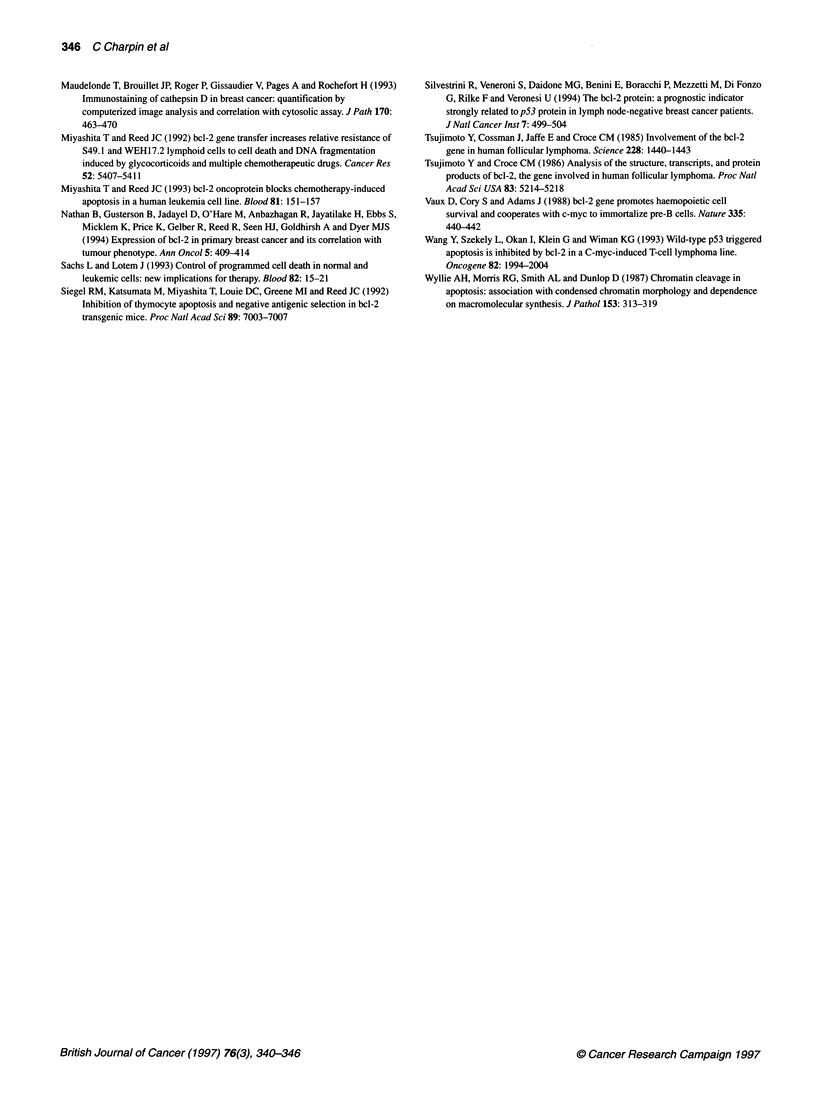# Automated and quantitative immunocytochemical assays of Bcl-2 protein in breast carcinomas.

**DOI:** 10.1038/bjc.1997.388

**Published:** 1997

**Authors:** C. Charpin, S. Garcia, C. Bouvier, B. Devictor, L. Andrac, M. N. Lavaut, C. Allasia

**Affiliations:** Department of Pathology, FacultÃ© de MÃ©decine Timone, Marseille, France.

## Abstract

**Images:**


British Journal of Cancer (1997) 76(3), 340–346
© 1997 Cancer Research Campaign

### Automated and quantitative immunocytochemical assays of Bcl-2 protein in breast carcinomas

C Charpin[1], S Garcia[2], C Bouvier[2], B Devictor[1], L Andrac[1], M-N Lavaut[1] and C Allasia[1]

[1]Department of Pathology, Faculté de Médecine Timone and [2]Department of Pathology, Hôpital Nord Marseilles, France

**Summary** Expression of the *bcl-2* gene was investigated in 218 human breast carcinomas by immunohistochemical analysis. Immunodetections were assessed using (1) frozen sections, (2) documented commercially available monoclonal antibody (bcl-2/124, Dako), (3) automation of immunoperoxidase technique (Ventana) and (4) quantitative evaluation of results by image analysis (SAMBA) and statistical analysis of quantitative data (BMDP software). Bcl-2 protein expression was correlated with current prognostic indicators and with molecular markers detected by the same procedure as for Bcl-2. It was shown that Bcl-2 expression is not related to patients' age, tumour size and type or lymph node status, but an inverse relationship was observed between Bcl-2 and tumour grade ($P < 0.0001$). An inverse relationship was also observed between Bcl-2 expression and p53 ($P < 0.0001$), Ki67/MIB1 antigen- ($P = 0.0012$), and P-gp- ($P = 0.002$) positive immunoreactions. In contrast, anti-Bcl-2 positive reaction was significantly associated with ER-positive ($P < 0.001$) and with ER/PR-positive or ER/PR/pS2-positive immunoreactions ($P \leq 0.005$). *Bcl-2* expression was independent of CD31 and cathepsin D expression. Thus, Bcl-2 protein, thought to be antiapoptotic, exhibits parodoxical expression in human breast carcinomas. It is strongly detected in low-grade tumours (well-differentiated) with low (MIB1) growth fraction, but is independent of the tumour progression (size, node status, CD31, and cathepsin D). Bcl-2 acting on apoptosis is related to *p53* gene abnormalities in breast carcinomas. Bcl-2 protein expression may also be involved in response to endocrine therapy (associated to ER/PR/pS2 positive immunoreactions) and probably with chemoresistance mechanisms (inverse relationship with P-gp).

**Keywords:** Bcl-2 protein; automated and quantitative immunohistochemistry; breast carcinoma

*Bcl-2* gene abnormality was first reported in non-Hodgkin's lymphomas (Tsujimoto et al, 1985), in which t(14;18) chromosomal translocation results in an inappropriately high level of *bcl-2* gene expression (Korsmeyer, 1992). The protein encoded by this gene has been shown to contribute to malignant cell expansion by prolonging cell survival (Vaux et al, 1988) and delaying or blocking programmed cell death/apoptosis (Hockenbery et al, 1990; Siegel et al, 1992). The human Bcl-2 protein is an intracellular membrane protein with a molecular mass of 24 kDa (Chen-Levy et al, 1989, 1990). It resides in the nuclear envelope, endoplasmic reticulum and outer mitochondrial membranes (Krajewski et al, 1993) with a non-uniform distribution suggestive of participation in protein complexes (Krajewski et al, 1993).

Apoptosis is an active form of cell death (Wyllie et al, 1987), requiring activation of endonucleases inducing the degradation of nuclear DNA of the cell into oligonucleosome-length fragments. Apoptosis plays an important role in normal physiological conditions, but in proliferative cells and tumours it may also be the target for chemotherapeutic drugs that can kill cancer cells by activating biochemical pathways leading to programmed cell death (Eastman et al, 1990). Drugs may act on the apoptotic process by inhibition of antiapoptotic factors up-regulated in malignant tumours. Chemoresistance may result from antiapoptotic protection from cell death and DNA fragmentation induced by antineoplastic drugs (Miyashita, 1992, 1993). In this regard high levels of Bcl-2 protein have been shown to protect lymphoid cells from antineoplastic drugs (Miyashita, 1992, 1993). In leukaemic cells, it can inhibit apoptosis induced by glucocorticoids (Alnemri et al, 1992) and 5-fluorodeoxyuridine or taxol (Fisher et al, 1993). Thus, detection of this protein in tissue may be informative for monitoring the therapy of patients with cancer. However, the true significance of Bcl-2 protein expression in tumours still remains to be investigated accurately, particularly in human breast carcinomas.

In breast carcinomas a high fraction of Bcl-2-positive cells correlates with good prognostic indicators (Silvestrini et al, 1994), longer survival in node-positive patients (Hellemans et al, 1995), and oestrogen receptor status (ER) (Alnemri et al, 1992; Johnston et al, 1994; Silvestrini et al, 1994; Hellemans et al, 1995; Baba et al, 1996). In addition, a highly significant relationship has been observed between the response to endocrine therapy and the presence of Bcl-2 protein in the tumour (Gee et al, 1994). Bcl-2 protein expression has been shown to be enhanced in ER-positive tumours after tamoxifen in association with reduced cell proliferation (Johnston et al, 1994). These results in breast carcinomas are somewhat paradoxical as it would be expected that Bcl-2 protein would counteract the tumour-inhibitory effect of endocrine therapy and that it would be down-regulated in tumours with good prognosis, with an inverse relationship with survival rates, as it is thought to prevent programmed cell death.

Only a few recent studies screening Bcl-2 protein expression in breast carcinomas have been reported (Silvestrini et al, 1994; Hellemans et al, 1995; Johnston et al, 1994; Baba et al, 1996), and in these reports methods of investigation were different. When

*Received 22 May 1996*
*Revised 20 September 1996*
*Accepted 27 November 1996*

*Correspondence to:* C Charpin, Laboratoire d'Anatomie Pathologique, EA 875 'Oncogénèse des Tumeurs Solides' – Faculté de Médecine Timone, 27, Bd Jean Moulin 13385 Marseille Cedex V, France immunohistochemistry was used to detect Bcl-2 protein in tissue sections from the tumours, samples were either fixed and paraffin embedded (Johnston et al, 1994; Silvestrini et al, 1994; Hellemans et al, 1995; Baba et al, 1996) or frozen (Gee et al, 1994). Antibodies directed against Bcl-2 protein were also different and variably diluted. Results were evaluated by semiquantitative analysis, which relies on the observers' subjectivity, with variable and arbitrary 'cut-off' points (Johnston et al, 1994; Hellemans et al, 1995). The rates of Bcl-2 protein-positive tumours were highly variable, ranging from 32% to 75% (Gee et al, 1994; Johnston et al, 1994; Silvestrini et al, 1994; Hellemans et al, 1995; Baba et al, 1996). As already mentioned (Johnston et al, 1994), validation of statistical analysis is difficult because of the lack of standardization of immunohistochemical assays and therefore practical conclusions, particularly with regard to the clinical significance of Bcl-2 expression in tissue, may not be clearly drawn from the literature survey.

In the present study, we have evaluated Bcl-2 protein expression in 218 breast carcinomas using immunohistochemical assays assessed in optimal technical conditions. Bcl-2 expression was detected (1) on frozen sections (with no risk of antigen damage owing to fixation and heating during paraffin embedding), (2) using a well-documented commercially available antibody (Dako/Bcl-2, 124), (3) by automated immunodetection (Ventana device) more reproducible than manual procedures and (4) by quantitative analysis of the results by densitometry of digitized coloured microscopic images (image analyser SAMBA). Bcl-2 expression in tumours was correlated with histological prognostic indicators and with expression of proteins also endowed with prognostic significance, such as growth fraction (detected by MIB1), p53, CD-31 (angiogenesis), cathepsin D (extracellular matrix protease) and hormone receptors and P-gp (multidrug resistance) evaluated according to the same procedure.

### MATERIAL AND METHODS

### Source of tissue samples

The specimens were surgically obtained from 218 patients with breast carcinomas from January 1993 to May 1994. Mean age was 56.7 years (range 32–83 years). For all patients surgical resection was the primary treatment and none received irradiation or chemotherapy preoperatively. Surgical specimens were fixed in Bouin's fixative, paraffin embedded and stained with haematoxylin, eosin and saffronin for routine microscopic diagnosis. Samples for immunodetection were taken, by pathologists, from the representative cancerous lesions, in the same area as the sample used for the intraoperative microscopic diagnostic assessed on frozen sections. Tissue samples for immunodetections were promptly dipped in liquid nitrogen and stored frozen at −80°C in the tumour library of our laboratory.

### Histopathological features

Tumour size ranged from 4 to 80 mm (mean = 16.8, s.d. = 11.95, median = 15 mm). In 90% of patients (198/218), axillary lymph node excision was performed; 62% ($n$ = 124) were node negative and 38% ($n$ = 75) were node positive.

Tumours were in situ in 17/218 cases (8%). Invasive ductal carcinomas accounted for 66% of cases (143/218), lobular carcinomas for 17% (38/218) and invasive carcinomas of other types for 9% (20/218).

Tumours were graded according to the Bloom grading system (SBR) (Bloom, 1957). Grade I tumours accounted for 22% (45/201), grade II tumours for 54% (108/201) and grade III tumours for 24% (48/201). Tumours were also graded according to a modified Bloom grading system (Le Doussal et al, 1989) into five grades: 8% of grade I, 30% of grade II, 31% of grade III, 27% of grade IV and 4% of grade V. Tumours were also ranked according to the Nottingham prognostic index (NPI) (Galea et al, 1992), which ranged from 2.1 to 7.3 (mean = 4.1, s.d. = 1.22).

### Immunohistological staining procedures

#### Antibodies sources

Monoclonal mouse anti-human Bcl-2 oncoprotein (Dako, *bcl-2/* 124, diluted 1 : 100) recognizes a peptide sequence comprising amino acids 41–54 of Bcl-2 protein (Clearly et al, 1986; Tsujimoto, 1986). The other monoclonal antibodies used were all commercially available and used as described previously (Charpin et al, 1988 *a–c*; 1993, 1994, 1995*a*, *b*; Charpin and Pellissier, 1994): MIB1 and anti-CD-31 (Immunotech, Marseille, France), anti-p53 clone DO-6 (Oncogene Science, Paris, France), anti-cathepsin D (CisBio International, Gif sur Yvette, France), anti-P-glycoprotein (P-gp) clone JSB1-N (Tebu, Le Perray en Yvelines, France), anti-oestrogen and progesterone receptors (ER/PR) (Abbott kits, Rungis, France).

#### Automated immunohistochemistry

Automated immunohistochemisty (immunoperoxidase) was performed on consecutive sections (4 μm thick) on Ventana 320 device (Grogan et al, 1993; 1995; Galaktionov et al, 1995) (Ventana Medical Systems, Tucson, AZ, USA) with Ventana kits (Ventana Medical Systems, Strasbourg, France) including also amino-ethyl carbazol reagent. Sections were counterstained with haematoxylin, dehydrated and mounted in glycergel.

### Image processing and statistical analysis

Immunostaining was analysed using an Axiophot microscope (Zeiss, Rueil Malmaison, France) and a 3 CCD camera (Sony,

**Figure 1** Immunohistochemical procedure using frozen sections, anti-Bcl-2 and 124 (Dako, 1:100) avidin–biotin–peroxidase (automated procedure/ Ventana). In invasive ductal carcinoma, the distribution of Bcl-2 protein is heterogeneous, involving not all the tumour cells and partly involving the cytoplasm adjacent to the cell membrane

© Cancer Research Campaign 1997

**Figure 2** Immunohistochemical procedure using frozen sections, anti-Bcl-2 and 124 (Dako, 1:100) avidin–biotin–peroxidase (automated procedure/ Ventana). High magnification shows that Bcl-2 protein is located in the cell, focally along cell membrane cytoplasm, and along nuclei (invasive lobular carcinoma)

**Figure 3** Immunohistochemical procedure using frozen sections, anti-Bcl-2 and 124 (Dako, 1:100) avidin–biotin–peroxidase (automated procedure/ Ventana), and quantitative image analysis (SAMBA). (**A**) Distribution of Bcl-2 immunostained surface (per cent of positive surface/counterstained surface). (**B**) Distribution of quantitative immunocytochemical index (per cent of stained surface/mean optimal density × 100) in the series investigated (*n* = 218 breast carcinomas)

Paris, France) and then processed by an image analysis system (SAMBA 2005, Alcatel-TITN, Grenoble, France) (Brugal et al, 1979). The two parameters of densitometric analysis, percentage of immunostained surface (vs counterstained surface) and mean optical density (MOD), which reflects the staining intensity (SAMBA arbitrary units scale ranging from 0 to 255), were obtained as previously reported (Charpin et al, 1988 *a–c*, 1994, 1995; Charpin and Pellissier, 1994). Statistical analysis was assessed using BMDP (Biomedical Data Package) software (University of California, Berkeley, CA, USA). Various statistical tests were used, depending upon the type (nominal or ordinal) and the distribution (normal or not) of the variables. Consequently, parametric or non-parametric tests were applied, including the chi-square test, Student's *t*-test, Kruskal–Wallis test, Mann–Whitney *U*-test, and the computation of correlation coefficients (Spearman, Kendall and Pearson). In addition, a quantitative index of immunoreactions (QIC) (Maudelonde et al, 1993), combining the percentage of stained surface and mean optical density (MOD), was computed (per cent of stained surface/MOD × 100).

### RESULTS

### Bcl-2 distribution patterns in cells and tissues

Patterns of immunoreaction are shown and described in detail in Figures 1 and 2. In brief, anti-Bcl-2/124 reacted only with epithelial tumour cells and not with lymphocytes or with stromal cells.

The positive immunoreactions with anti-Bcl-2 did not differ in ductal carcinomas, lobular carcinomas or carcinomas of other types and was observed in cell cytoplasm focally along the cell membrane.

In intraductal carcinomas or in intraductal component of invasive carcinomas the positive Bcl-2 immunostaining exhibited the same pattern as seen in invasive areas. In normal breast present along tumour borders, a positive Bcl-2 reaction was focally observed.

### Bcl-2 quantitative immunodetection

Among the 218 tumours probed, 171 (78%) were Bcl-2 positive and 47 were Bcl-2 negative. Distribution of anti-Bcl-2 positive staining evaluated by densitometry on tissue sections is shown on Figure 3A and B. In positive tumours the tumour surface stained by anti-Bcl-2 ranged from 3% to 70% (mean = 22.4%, s.d. = 12.1) (Figure 3A). The Bcl-2 quantitative immunocytochemical index (QIC) varied from 2.6 to 49.2 (mean = 15.2, s.d. = 9.8) (Figure 3B).

### Bcl-2 expression and clinicopathological data

Bcl-2-immunostained surface evaluated by image analysis and QIC was independent of the patients' age, the tumour size, the histological type, lymph node status and the NPI.

In contrast, an inverse relationship was observed between Bcl-2 expression and tumour grades (Table 1), with a significant ($P < 0.001$) decrease in Bcl-2 expression in high-grade tumours, suggesting that Bcl-2 protein is down-regulated in poorly differentiated tumours.

### Bcl-2 expression and quantitative immunodetection of other molecular markers

Results of quantitative evaluation of anti-ER and PR antigenic sites and anti-pS2, of growth fraction (MIB1), and also of

© Cancer Research Campaign 1997

**Table 1** Correlation of Bcl-2 expression and tumour grades (218 breast carcinomas)

| Tumour grades | Bcl-2 immunohistochemical expression | | | |
|---|---|---|---|---|
| | Bcl-2 negative ($n$ = 43) | | Bcl-2 positive (% stained surface) | |
| Grade (Bloom) | | | | |
| 1 | 5 (12%) | | 27.4% ± 11.5 | |
| 2 | 13 (30%) | $P$ < 0.001[a] | 22.3% ± 9.3 | $P$ = 0.011[b] |
| 3 | 25 (58%) | | 5.8% ± 7.2 | |
| Grade modified (Le Doussal) | | | | |
| 1 + 2 (group I) | 8 (18%) | | 24.7% ± 10.5 | |
| 3 (group II) | 10 (23%) | $P$ < 0.001[a] | 20.5% ± 8.4 | $P$ = 0.0043[b] |
| 4+5 (group III) | 25 (59%) | | 14.3% ± 5.4 | |

[a]Chi-square. [b]Kruskal–Wallis.

**Table 2** Correlation of Bcl-2 protein (anti bcl-2/124, Dako) and oestrogen and progesterone receptor antigenic sites (ER, PR) and P-S2 detected by automated (Ventana) and quantitative (SAMBA) immunohistochemistry in 218 breast carcinomas.

| | Bcl-2 immunoreaction | | | |
|---|---|---|---|---|
| | Positive surface (%) | | QIC[a] | |
| ER | | | | |
| Positive | 24 (±10) | $P^b$ = 0.004 | 16 (±10) | $P^b$ = 0.005 |
| Negative | 14 (±8) | | 11 (±6) | |
| ER/PR | | | | |
| Positive | 25 (±13) | $P^b$ = 0.005 | 19 (±12) | $P^b$ = 0.0018 |
| Negative | 11 (±9) | | 12 (±10) | |
| ER/PR/pS2 | | | | |
| Positive | 24 (±16) | $P^b$ = 0.001 | | NS[b] |
| Negative | 11 (±9) | | | |

[a]Quantitative immunocytochemical index. [b]Mann–Whitney test.

**Table 3** Correlation of Bcl-2 protein expression (anti bcl-2/124, Dako) to cathepsin D CD31, MIB1, P53, P-glycoprotein in 218 breast carcinomas. All antigens were detected by immunohistochemistry on frozen sections, automated procedures (Ventana) and quantitative analysis (SAMBA image analyser)

| | Bcl-2 immunoreaction | | Bcl-2 positive surface |
|---|---|---|---|
| | Positive | Negative | (%) |
| *p53* | | | |
| Positive | 23/51 (45.1%) | 28/51 (54.9%) | $r^a$ = – 0.28 $P$ < 0.0001 |
| Negative | 142/166 (85.5%) | 24/166 (14.4%) | |
| | $P^b$ < 0.0001 | | |
| *P-glycoprotein* | | | |
| Positive | 62/169 (37%) | 29/47 (62%) | $r^a$ = – 0.34 $P$ < 0.0001 |
| Negative | 107/169 (63%) | 18/47 (38%) | |
| | $P^b$ < 0.01 | | |
| *Cathepsin D positive* | | | NS |
| *CD 31* positive | | | NS |
| *MIB1* positive (Ki67) | | | $r^a$ = – 0.30 $P$ < 0.0001 |

[a]Pearson's coefficent correlation. [b]Chi-square.

© Cancer Research Campaign 1997

immunoreactions with anti-p53, anti-cathepsin D, anti-CD-31 and anti-P-gp are shown in Tables 2 and 3. A significant correlation was observed between Bcl-2 protein expression and the tumour tissue contents of ER, PR and pS2 antigens, evaluated by the same procedure on consecutive frozen sections from the same tissue blocks (Table 2). An inverse relationship was observed between Bcl-2 and p53 ($P < 0.001$) and growth fraction (MIB1/Ki67) ($P < 0.0001$) and P-gp ($P = 0.0021$) expression in tumours (Table 3). In contrast, Bcl-2 was unrelated to CD31 (reflecting stromal angiogenesis) and to cathepsin D (tumour cell secretion of extracellular matrix protease) (Table 3).

### DISCUSSION

In these series, the Bcl-2 protein was detected in 78% of breast carcinomas by automated and quantitative immunohistochemistry using frozen samples and Bcl-2/124 MAb. In previous reports, Bcl-2 protein was detected in 32–75% of cases (Gee et al, 1994; Johnston et al, 1994; Leek et al, 1994; Nathan et al, 1994; Silvestrini et al, 1994; Hellemans et al, 1995; Baba et al, 1996). The results obtained in these different studies cannot be compared because of variations in the methods of detection. However, detection on frozen samples avoids bias resulting from antigen damage because of fixatives, uncontrolled fixation duration or uncontrolled variations in antigen heating during paraffin embedding. Also, the results rely upon the quality of the technique (Charpin et al, 1994*b*), and ideally automation provides for a better quality control (Grogan et al, 1993, 1995; Galaktionov et al, 1995), particularly with regard to reproducibility, in addition to the fact that many tissue samples may be identically probed at the same time.

Similarly, shortcomings may also result from semiquantitative analysis of immunostaining. Semiquantitative evaluation of results of immunodetection is very convenient, rapidly realized and is an inexpensive method of analysis. However, its reliability depends upon observer experience. Although semiquantitative analysis is sufficient to evaluate negative vs positive reactions, or very weak vs very strong immunoreactions, it is not sufficiently accurate to evaluate quantitatively intermediate patterns of staining. This must be pointed out, particularly when quantitative variations in the distribution of antigen are correlated with prognostic parameters such as survival, metastasis and recurrence or various histoprognostic indicators.

Discrepancies in the prognostic significance of Bcl-2 protein expression in major human carcinomas probably result from this type of analysis bias. In the present study we evaluated the results of Bcl-2 protein expression in tissue by computerized processing of digitized microscopic image, using the same hardware and software as in previous studies (Charpin et al, 1988 *a–c*, 1993, 1994*a*, *b*), 1995*a*, *b*). The results were obtained more objectively, variations of staining more accurately evaluated and numerical values of parameters were more appropriate to statistical analysis (continuous variables).

We observed that Bcl-2 protein expression was greater in low-grade than in high-grade tumours when the Bloom grading system (Bloom, 1957) or a modified system (Le Doussal et al, 1989) was employed. In these respects our results are not in agreement with those of others (Hellemans et al, 1995; Nathan et al, 1994).

In contrast, like others (Hellemans et al, 1995), we found no relationship between Bcl-2 protein expression and tumour size and the axillary lymph node status.

The prognostic significance of Bcl-2 expression in terms of relapse-free survival and overall survival has been evaluated in some recent studies (Nathan et al, 1994; Silvestrini et al, 1994; Hellemans et al, 1995), using immunohistochemistry of archival fixed tissue samples, and provided discrepant results. Bcl-2 protein expression was not significantly related to a better or worse survival (disease-free survival and overall survival) in the study of Nathan et al (1994). In contrast, in the study by Hellemans et al (1995), although no prognostic value was demonstrated for Bcl-2 protein expression on disease-free survival and overall survival in node-negative breast cancer patients, in node-positive patients Bcl-2 expression was independently related to shortened survival (disease-free and overall survival) using multivariate analysis. In the Silvestrini et al study (1994) Bcl-2 expression in node-negative patients was related to better 6-year survival, but this predictive role of Bcl-2 protein expression was mainly dependent on *p53* expression. Thus, it appears that the prognostic significance of Bcl-2 expression in breast carcinomas deserves additional investigations, in larger series, with longer follow-up, and more standardized immunohistochemical assays (work in preparation).

We compared the quantitative evaluation of Bcl-2 expression in tissue with tumour growth fraction as evaluated by MIB1 MAb. Previous reports have shown discrepant results, demonstrating a non-relationship between Ki67-positive immunoreaction and Bcl-2 protein expression (Gee et al, 1994) or an inverse relationship (Johnston et al, 1994). In our series we also observed an inverse relationship between growth fraction and Bcl-2 expression ($P < 0.0001$).

In contrast, we observed no relationship between Bcl-2 expression and anti-CD-31 (Charpin et al, 1995*b*) or anti-cathepsin D (Charpin et al, 1993; Maudelonde et al, 1993) positive immunoreactions, suggesting that Bcl-2 involvement in the apoptotic process is independent of angiogenesis and protease synthesis, acting on extracellular matrix digestion. These results also suggest that Bcl-2 is independent of mechanisms of tumour progression.

It has been shown that the *bcl-2* gene can inhibit apoptosis triggered by the wild-type *p53* gene (Wang et al, 1993). In contrast, the mutant *p53* gene can inhibit apoptosis (Lotem, 1993) although it does not seem to have the same large protective range as *bcl-2* (Sachs, 1993). An inverse correlation between the expression of Bcl-2 protein and mutant p53 was observed in the MCF 7 breast cancer cell line (Haldar et al, 1994) and in tissue sections of breast carcinomas (Baba et al, 1996; Silvestrini et al, 1994), as in our study, suggesting that mutant *p53* could substitute for *bcl-2* function in breast cancer cells and that it could also down-regulate Bcl-2 expression (Haldar et al, 1994).

We observed an inverse relationship between Bcl-2 and P-gp expression that is related to multidrug resistance. These results are also paradoxical as Bcl-2 protein has been shown to be involved in chemoresistance (Alnemri et al, 1992; Miyashita, 1992; 1993; Fisher et al, 1993).

Immunostaining of steroid hormone receptors has been shown to be strongly associated with that of Bcl-2 protein (Gee et al, 1994; Johnston et al, 1994; Nathan et al, 1994; Silvestrini et al, 1994; Hellemans et al, 1995; Baba et al, 1996), suggesting that this protein is under oestrogen regulation via oestrogen receptors (Gee et al, 1994). In this respect, the increased expression of Bcl-2 protein in ER-positive tumours of patients treated by tamoxifen shows that Bcl-2 protein expression can be modulated by therapy with anti-oestrogens (Johnston et al, 1994). We observed that Bcl-2 expression was significantly associated with ER-positive

 © Cancer Research Campaign 1997

detection in tumours ($P \leq 0.001$) and with $ER^+/PR^+/pS2^+$ ($P \leq 0.001$). These results suggest that Bcl-2 protein is probably involved in the response to endocrine therapy. This is unexpected, as mentioned above, as Bcl-2 is thought to prevent programmed cell death and should counteract the tumour-inhibitory effects of endocrine therapy. Indeed, patients whose tumours have strong Bcl-2 immunostaining appear to derive the greatest benefit from endocrine therapy (Gee et al, 1994).

The role of *bcl-2* and the significance of Bcl-2 protein in breast carcinomas remains to be elucidated, in particular it remains to be seen whether Bcl-2 expression is involved directly in the pathogenesis of breast carcinomas or, alternatively, whether it is induced secondarily by other genomic changes in tumour cells responsible for modulation of Bcl-2 protein expression. Our results suggest that Bcl-2 protein is related to tumour cell differentiation (grade and hormone receptors), independent of tumour progression (node status, tumour size, stromal angiogenesis and tumour proteasic activity). The potential role of Bcl-2 expression in patient response to endocrine therapy or chemotherapy emphasizes the clinical relevance of Bcl-2 protein immunodetection in tumours. However, the use of Bcl-2 immunohistochemical assays in the monitoring of patient therapy requires optimal technical conditions of detection that can be obtained in automated and quantitative (image analysis) immunohistochemical procedures using frozen tumour samples and well-documented monoclonal antibodies.

### ACKNOWLEDGEMENTS

This study was supported by grants from LNLCC (Ligues Départementales pour la Lutte contre le Cancer) and Fondation de France and Institut Fédératif de Recherche en Cancérologie et Immunologie de Marseille, Aix-Marseille University.

### REFERENCES

Alnemri ES, Fernandes TF, Haldar S, Croce CM and Litwack G (1992) Involvement of bcl-2 in glucocorticoid-induced apoptosis of human pre-B leukemias. *Cancer Res* **52**: 491–495

Baba M, Hideshima T, Shinohara T, Yamashita JI and Shirakusa T (1996) Immunohistochemical analysis of bcl-2 and p53 protein in breast carcinoma. *Int J Oncol* **8**: 355–358

Bloom HJG and Richardson WW (1957) Histological grading and prognosis in breast cancer. *Br J Cancer* **11**: 359–377

Brugal G, Garbay C, Giroud F and Adhel D (1979) A double scanning microphotometer for image analysis: hardware, sofware and biomedical application. *J Histochem Cytochem* **27**: 144

Charpin C and Pellissier JF (1994) Marqueurs moléculaires dans les cancers du sein: aspect pratique et évaluation morphologique. *Bull Acad Natl Med* **178**: 475–493

Charpin C, Andrac L, Vacheret H, Habib MC, De Victor B, Lavaut MN and Toga M (1988*a*) Multiparametric evaluation (SAMBA) of growth fraction (monoclonal Ki67) in breast carcinoma: a correlation of immunodetection in tissue sections and morphological data and estrogen and progesterone receptor immunocytochemical assays. *Cancer Res* **48**: 4368–4374

Charpin C, Jacquemier J, Andrac L, Vacheret H, Habib MC, DeVictor B and Toga M (1988*b*) Multiparametric analysis (SAMBA 200) of the progesterone receptor immunocytochemical assay in non malignant and malignant breast isorders. *Am J Pathol* **132**: 199–211

Charpin C, Martin P, Devictor B, Lavaut MN, Habib MC and Toga M (1988*c*) Multiparametric study (SAMBA 200) of estrogen receptor immunocytochemical assay in 400 human breast carcinomas analysis of estrogen receptor distribution heterogeneity in tissues and correlations with dextran coated charcoal assays and morphological data. *Cancer Res* **48**: 1578–1586

Charpin C, Devictor B, Bonnier P, Andrac L, Lavaut MN, Allasia C and Piana L (1993) Cathepsin D immunocytochemical assays in breast carcinomas: image analysis and correlation to prognostic factors. *J Path* **170**: 463–470

Charpin C, Vielh P, Duffaud F, Devictor B, Andrac L, Lavaut MN, Allasia C, Horschowski N and Piana L (1994) Quantitative immunocytochemical assays of P-glycoprotein in breast carcinomas: correlation to messenger RNA expression and to immunohistochemical prognostic indicators. *J Natl Cancer Inst* **86**: 1539–1545

Charpin C, Devictor B, Andrac L, Amabile J, Bergeret D, Lavaut MN, Allasia C and Piana L (1995*a*) P-53 quantitative immunocytochemical analysis in breast carcinomas. *Hum Pathol* **26**: 159–166

Charpin C, Devictor B, Bergeret D, Andrac L, Boulat J, Horschowski N, Lavaut MN and Piana L (1995*b*) CD 31 quantitative immunohistochemical assays in breast carcinomas. Correlation with current prognostic factors. *Am J Clin Path* **103**: 443–448

Chen-Levy S and Cleary ML (1990) Membrane topology of the bcl-2 proto-oncogenic protein demonstrated in vitro. *J Biol Chem* **265**: 4929–4933

Clearly ML, Smith SD and Sklar J (1986) Cloning and structural analysis of cDNAs for bcl-2 and a hybrid bcl-2/immunoglobulin transcript resulting from the t (14;18) translocation. *Cell* **47**: 19–28

Chen-Levy Z, Nourse J and Cleary MZ (1989) The bcl-2 candidate proto-oncogene product is a 24-kD integral membrane protein highly expressed in lymphoid cell lines and lymphomas carrying the t [14;18] translocation. *Mol Cell Biol* **9**: 701–710

Eastman A (1990) Activation of programmed cell death by anticancer agents: cisplatin as a model system. *Cancer Cells* **2**: 275–280

Fisher TC, Milner AE, Gregory CD, Jackman AZ, Wynne-Aherne G and Hartley JA (1993) bcl-2 modulation of apoptosis induced by anticancer drugs: resistance to thymidylate stress is independent of classical resistance pathways. *Cancer Res* **53**: 3321–3327

Galaktionov K, Lee AK, Eckstein J, Draetta G, Meckler J, Loda M and Beach D (1995) CDC25 Phosphatases as potential human oncogenes. *Science* **269**: 1575–1577

Galea MH, Blamey RW, Elston CE and Ellis IO (1992) The Nottingham prognostic index in primary breast cancer. *Br Cancer Res Treat* **22**: 207–219

Gee JMW, Robertson JFR, Ellis IO, Willsher P, McClelland RA, Hoyle HB, Kyme SR, Finlay P. Blamey RW and Nicholson RI (1994) Immunocytochemical localization of bcl-2 protein in human breast cancers and its relationship to a series of prognostic markers and response to endocrine therapy. *Int J Cancer* **59**: 619–628

Grogan TM, Casey TT, Miller PC, Rangel CS, Nunnery DW and Naglo RB (1993) Automation of immunohistochemistry. *Adv Path Lab Med* **6**: 253–283

Grogan TM, Rangel C, Rimsza L, Bellamy W, Martel R, McDaniel D, McGraw B, Richards W, Richter L, Rodgers P, Rybski J, Showalter W, Vela E and Zeheb R (1995) Kinetic-Mode, automated double-labeled immunohistochemistry and in situ hybridation in diagnostic pathology. *Adv Path Lab Med* **8**: 79–99

Haldar S, Negrini M, Monne M, Sabbioni S and Croce CM (1994) Down-regulation of bcl-2 by *p53* in breast cancer cells. *Cancer Res* **54**: 2095–2097

Hellemans P, Van Dam PA, Weyler J, Van Oosterom AT, Buytaert P and Van Marck E (1995) Prognostic value of bcl-2 expression in invasive breast cancer. *Br J Cancer* **72**: 354–360

Hockenbery D, Nunez G, Milliman C, Schreiber RD and Korsmeyer SJ (1990) bcl-2 is an inner mitochondrial membrane protein that blocks programmed cell death. *Nature* **348**: 334–336

Johnston SRD, MacLennan KA, Sacks NPM, Salter J, Smith IE and Dowsett M (1994) Modulation of bcl-2 and Ki-67 expression in oestrogen receptor-positive human breast cancer by tamoxifen. *Eur J Cancer* **11**: 1662–1669

Korsmeyer SJ (1992) bcl-2 initiates a new category of oncogene regulators of cell death. *Blood* **80**: 879–886

Krajewski S, Tanaka S, Takayama S, Schibler MJ, Fenton W and Reed JC (1993) Investigation of the subcellular distribution of the bcl-2 oncoprotein: Residence in the nuclear envelope, endoplasmic reticulum, and outer mitochondrial membranes. *Cancer Res* **53**: 4701–4714

Le Doussal V, Tubiana-Hulin M, Friedman S, Hacene K, Spyratos F and Brunet M (1989) Prognostic value of histologic grade nuclear components of Scarff–Bloom–Richardson (SBR). An improved score modification based on a multivariated analysis of 1262 invasive ductal breast carcinomas. *Cancer* **64**: 1914–1921

Leek RD, Kaklamanis L, Pezzella F, Gatter KC and Harris AL (1994) bcl-2 in normal human breast and carcinoma, association with oestrogen receptor-positive, epidermal growth factor receptor-negative tumours and *in situ* cancer. *Br J Cancer* **69**: 135–139

Lotem J and Sachs L (1993) Regulation by bcl-2, myc, and *p53* of susceptibility to induction of apoptosis by heat shock and cancer chemotherapy compounds in differentiation competent and detective myeloid leukemic cells. *Cell Growth Different* **4**: 41–47

© Cancer Research Campaign 1997

Maudelonde T, Brouillet JP, Roger P, Gissaudier V, Pages A and Rochefort H (1993) Immunostaining of cathepsin D in breast cancer: quantification by computerized image analysis and correlation with cytosolic assay. *J Path* **170**: 463–470

Miyashita T and Reed JC (1992) bcl-2 gene transfer increases relative resistance of S49.1 and WEH17.2 lymphoid cells to cell death and DNA fragmentation induced by glycocorticoids and multiple chemotherapeutic drugs. *Cancer Res* **52**: 5407–5411

Miyashita T and Reed JC (1993) bcl-2 oncoprotein blocks chemotherapy-induced apoptosis in a human leukemia cell line. *Blood* **81**: 151–157

Nathan B, Gusterson B, Jadayel D, O'Hare M, Anbazhagan R, Jayatilake H, Ebbs S, Micklem K, Price K, Gelber R, Reed R, Seen HJ, Goldhirsh A and Dyer MJS (1994) Expression of bcl-2 in primary breast cancer and its correlation with tumour phenotype. *Ann Oncol* **5**: 409–414

Sachs L and Lotem J (1993) Control of programmed cell death in normal and leukemic cells: new implications for therapy. *Blood* **82**: 15–21

Siegel RM, Katsumata M, Miyashita T, Louie DC, Greene MI and Reed JC (1992) Inhibition of thymocyte apoptosis and negative antigenic selection in bcl-2 transgenic mice. *Proc Natl Acad Sci* **89**: 7003–7007

Silvestrini R, Veneroni S, Daidone MG, Benini E, Boracchi P, Mezzetti M, Di Fonzo G, Rilke F and Veronesi U (1994) The bcl-2 protein: a prognostic indicator strongly related to *p53* protein in lymph node-negative breast cancer patients. *J Natl Cancer Inst* **7**: 499–504

Tsujimoto Y, Cossman J, Jaffe E and Croce CM (1985) Involvement of the bcl-2 gene in human follicular lymphoma. *Science* **228**: 1440–1443

Tsujimoto Y and Croce CM (1986) Analysis of the structure, transcripts, and protein products of bcl-2, the gene involved in human follicular lymphoma. *Proc Natl Acad Sci USA* **83**: 5214–5218

Vaux D, Cory S and Adams J (1988) bcl-2 gene promotes haemopoietic cell survival and cooperates with c-myc to immortalize pre-B cells. *Nature* **335**: 440–442

Wang Y, Szekely L, Okan I, Klein G and Wiman KG (1993) Wild-type p53 triggered apoptosis is inhibited by bcl-2 in a C-myc-induced T-cell lymphoma line. *Oncogene* **82**: 1994–2004

Wyllie AH, Morris RG, Smith AL and Dunlop D (1987) Chromatin cleavage in apoptosis: association with condensed chromatin morphology and dependence on macromolecular synthesis. *J Pathol* **153**: 313–319

© Cancer Research Campaign 1997